# Immature oocyte incidence: Contributing factors and effects on mature sibling oocytes in intracytoplasmic sperm injection cycles

**DOI:** 10.5935/1518-0557.20190056

**Published:** 2020

**Authors:** Daniela Paes de Almeida Ferreira Braga, Bianca Ferrarini Zanetti, Amanda Souza Setti, Assumpto Iaconelli Jr, Edson Borges Jr.

**Affiliations:** 1 Fertility Medical Group, São Paulo, SP - Brazil; 2 Instituto Sapientiae - Centro de Estudos e Pesquisa em Reprodução Humana Assistida, São Paulo, SP - Brazil

**Keywords:** germinal vesicle, metaphase I, immature oocyte, ICSI, pregnancy

## Abstract

**Objective:**

The aim of this study was to investigate which factors contribute to the incidence of immature oocytes (germinal vesicle -GV- and metaphase I -MI-) and how they impact the intracytoplasmic sperm injection (ICSI) outcomes of sibling mature oocytes.

**Methods:**

Data from 3,920 cycles performed from June/2010 to August/2016 in a private university-affiliated IVF center were evaluated for the influence of controlled ovarian stimulation protocol (COS) on immature oocytes incidence and its effects on ICSI outcomes.

**Results:**

MI (*p*=0.004) and GV (*p*=0.029) number were negatively correlated with gonadotropin dose. Patients stimulated by rFSH had increased GV/oocyte rate in both GnRH agonists (*p*<0.001) and antagonist (*p*=0.042) protocols, in comparison to rFSH associated with rLH protocol. MI and GV/oocyte rates were negatively correlated to fertilization (*p*<0.001), high-quality embryo on da *p*<0.001; GV/oocyte *p*=0.033) and pregnancy (MI/oocyte *p*=0.002; GV/oocyte *p*=0.013) rates. Cycles above a 10.5% MI/oocyte cut-off were correlated to higher response to ovarian stimulation, poor embryo development and almost two times lower pregnancy rate. Immature oocyte incidence is affected by COS and impacts on ICSI outcomes.

**Conclusion:**

Our evidence suggests that oocytes derived from a cohort with high incidence of maturation fail may have detrimental clinical outcomes.

## INTRODUCTION

By the time of birth, the human ovary contains a pool of quiescent primordial follicles, each consisting of a small inactive oocyte, arrested in the germinal vesicle (GV) stage, and a single layer of granulosa cells; the fate of each follicle is controlled by endocrine as well as paracrine factors (Findlay *et al*., 2015; Jaffe & Egbert, 2017). Of the millions of the primordial oocytes present at birth, only approximately 400 mature during a woman's lifetime (Monniaux *et al*., 2014). Oocyte maturation is defined as the restart and completion of the first meiotic division from prophase I, through metaphase I (MI stage), to metaphase II (MII stage), with accompanying cytoplasmic maturation, which includes the storage of cytoplasmic enzymes, mRNAs, organelles, and metabolic substrates that are crucial for fertilization and early embryonic development (Coticchio *et al*., 2015; Downs, 2015).

In contrast to the *in vivo* process, where oocyte maturation occurs as the result of the natural selection, in assisted reproduction cycles, supra-physiologic gonadotropin doses induce multiple follicular growth and maturation, in order to ensure the maximum number of obtained embryos, and thereafter the highest probability of a successful pregnancy (Bosch & Ezcurra, 2011; Fatemi *et al*., 2012). Even though controlled ovarian stimulation (COS) has been developed and refined in attempt to obtain optimal oocytes number from each cycle, different COS protocols may result in follicular asynchrony and variations in oocyte number, quality, viability, and competence (Jungheim *et al*., 2015).

Usually, 10 to 30% of oocytes are still immature at retrieval (GV or MI stages) (Borges *et al*., 2016; 2017). Some of the immature oocytes may extrude the first polar body and progress to maturity during in vitro culture, and might be considered for sperm injection, while others, especially those at the GV stage, will not mature during the observation time (Shu *et al*., 2007; Braga *et al*., 2010).

The asynchronous ovarian response, with numerous immature oocytes at ovum pick-up, can be an indicative that overall ovarian follicles were less responsive to ovarian stimulation, and that oocytes considered mature in the same cohort may not be fully competent for fertilization and embryo formation (Devreker *et al*., 1999; Halvaei *et al*., 2012). It has been suggested that maturation fail may lead to anomalies such as multinucleation and aneuploidy, which directly impacts the developmental competence of embryos (Nogueira *et al*., 2000; Emery *et al*., 2005).

As there are no macroscopic markers and no single observable factor indicating cytoplasmic maturation completion, there are scarce data about the probable impact of higher immature oocytes incidence in the developmental competence of mature oocytes from same cohort. Therefore, the goal for the present study was to investigate which factors contribute to the incidence of immature oocytes and how immature oocytes impacts on the outcomes of mature oocytes from the same cohort.

## MATERIALS AND METHODS

### Experimental design

This historical cohort study included data from 26,040 oocytes obtained from 3,920 cycles performed from June/2010 to August/2016, in a private university-affiliated IVF center. The inclusion criterion was couples undergoing first COS and ICSI cycle with fresh embryo transfer at day five. The exclusion criteria were as follows: ICSI cycle with vitrified/thawed or donated oocytes, surgical sperm retrieval, vitrified/thawed embryo transfer, or pre-implantation genetic testing.

The influence of COS protocol, pituitary suppression protocol, estradiol level on the day of hCG trigger, and interval between hCG trigger and oocyte retrieval on the numbers and rates of immature oocytes were evaluated.

The effects of immature oocytes rates on fertilization rate, high-quality embryos rates on cleavage-stage (days two and three), blastocyst rate, implantation rate, pregnancy rate and miscarriage rate were also analyzed.

All patients signed a written informed consent form and the study was approved by the local institutional review board.

### Controlled ovarian stimulation

COS was achieved by the administration of daily doses of recombinant FSH (Gonal-F^®^, Merck KGaA, Geneva, Switzerland); or recombinant FSH associated with recombinant LH (Gonal-F^®^ and Pergoveris^®^, Merck KGaA) beginning on day three of the cycle. Pituitary suppression was performed by GnRH antagonist (Cetrotide^®^; Merck KGaA) beginning when at least one follicle ≥14 mm was visualized, or GnRH agonist (Lupron Kit(tm), Abbott Laboratoires, Paris, France), from day 21 of the anterior menstrual cycle.

Follicular growth was monitored by transvaginal ultrasound examination. When ≥3 follicles attained a mean diameter of ≥17 mm and adequate serum estradiol levels were observed, recombinant hCG (Ovidrel^®^, Serono) was administered to trigger final follicular maturation. Ovum pick-up through transvaginal ultrasound was scheduled for 34-36 hours after hCG administration. The interval time between hCG administration and beginning of follicular aspiration was annotated. Oocyte yield was defined as the number of retrieved oocytes by the number of aspirated follicles.

### Oocyte preparation

Retrieved oocytes were maintained in culture media (Global for fertilization, LifeGlobal, Guilford, USA) supplemented with 10% protein (LGPS, LifeGlobal) and covered with paraffin oil (Paraffin oil P.G., LifeGlobal) for 4 h before cumulus cell removal. The cumulus cells were removed after exposure to a HEPES-buffered medium containing hyaluronidase (80 IU/ml, LifeGlobal). The remaining cells were mechanically removed by a hand-drawn Pasteur pipette (Humagen Fertility Diagnostics, Charlottesville, USA).

Oocyte morphology and maturation stage were assessed using an inverted Nikon Diaphot microscope with a Hoffmann modulation contrast system under 400x magnification (Eclipse TE 300 microscope, Nikon, Tokyo, Japan), just before sperm injection (5-7 hours after retrieval). Oocytes that had released the first polar body were considered mature (MII stage) and were submitted to ICSI.

Immature oocyte rates were defined by cycle as the number of MI oocytes by the total number of retrieved oocytes (MI/oocyte) and as the as number of GV oocytes by the total number of retrieved oocytes (GV/oocyte).

### Intracytoplasmic sperm injection

Intracytoplasmic sperm injection was performed according to Palermo *et al*. (1992). Fertilization was confirmed approximately 16 h after ICSI. Embryos were maintained in a 50-µL drop of culture medium (Global^®^, LifeGlobal) with 10% protein supplement and covered with paraffin oil in a humidified atmosphere under 6% CO_2_ at 37ºC for five days.

### Embryo quality and embryo transfer

Cleavage-stage morphology on days two and three were evaluated according to Istanbul consensus (Alpha Scientists in Reproductive Medicine & ESHRE Special Interest Group of Embryology, 2011). High-quality cleavage-stage embryos were defined as those with all of the following characteristics: three to five cells on day two or eight to ten cells on day three, <10% fragmentation, symmetric blastomeres, absence of multinucleation, colorless cytoplasm with moderate granulation and no inclusions, the absence of perivitelline space granularity and the absence of zona pellucida dimorphisms. Embryos lacking any of these characteristics were considered to be of low quality. The blastocyst rate was defined as the number of embryos that reached blastocyst stage at day five by the number of 2PN embryos.

Embryo transfer was performed on the fifth day of development using a soft catheter with transabdominal ultrasound guidance. Considering their morphology, one to two embryos were transferred per patient.

### Clinical follow-up

A pregnancy test was performed 10 days after embryo transfer. All women with a positive test received a transvaginal ultrasound scan after two weeks. Clinical pregnancy was diagnosed when fetal heartbeat was detected. Implantation rates were calculated per patient as the number of gestational sacs by the number of transferred embryos. Pregnancy rates were calculated per embryo transfer. Miscarriage was defined as a pregnancy loss before 20 weeks.

### Data analysis

The sample size calculation using G*Power 3.1.7 (Franz Faul, Universität Kiel, Germany) suggested that 2,848 cycles would be enough to demonstrate a 10% effect with 95% power and 5% significance level. The analysis was performed using SPSS Statistics 21 (IBM, New York, New York, USA).

The correlation between GV/oocyte and MI/oocyte rates was evaluated by Pearson ^®^.

To assess the contributing factors to immature oocyte incidence, total gonadotropin dose administered, estradiol level on the day of hCG administration, and interval between hCG trigger and oocyte retrieval were used as predictors in linear regression models. The models were adjusted for maternal age and maternal body mass index (BMI). The effects of COS protocol (rFSH *vs.* rFSH plus rLH) and pituitary suppression (GnRH agonist *vs.* antagonist) were evaluated by Generalized Linear Model followed by Bonferroni post hoc, adjusted for maternal age, maternal BMI and total gonadotropin dose.

To assess the correlation of immature oocyte rates and ICSI outcomes of mature oocytes from the same cohort, laboratorial outcomes (fertilization rate, high-quality embryos rates on day two and three, and blastocyst rate) were analyzed by linear regression models adjusted for maternal age, maternal BMI, total FSH dose, estradiol level on the day of hCG administration and number of retrieved oocytes. The correlation of immature oocyte rates and clinical outcomes (implantation, pregnancy, and miscarriage rates) were assessed by linear and binary logistic regression models adjusted for maternal age, maternal BMI, total FSH dose, estradiol level on the day of hCG administration, number of retrieved oocytes, number of transferred embryos and endometrial thickness.

Discriminant Analysis for pregnancy outcome prediction (positive vs. negative) was performed using as independent variables: MI/oocyte and GV/oocyte rates, maternal age, maternal BMI, total FSH dose, estradiol level on the day of hCG administration, number of retrieved oocytes, number of transferred embryos, and endometrial thickness. The data was grouped according with established cut-off for MI/oocyte rate, and the analysis were performed by Generalized Linear Model followed by Bonferroni post hoc, adjusted for the same confounders variables described above.

## RESULTS

The descriptions of patients' characteristics and ICSI outcomes are shown in Table 1. Although statistically significant, the correlation coefficient between GV/oocyte and MI/oocyte rates was low (Pearson's r= -0.079 *p*<0.001), therefore each rate was evaluated separately.

**Table 1:** Descriptive analyses of patients’ demographics and ICSI outcomes (n=3,920)

	Mean ± SD
***Patient’s demographics***	
Maternal age (years)	35.99±4.68
Maternal BMI (kg/m^2^)	24.38±3.87
Paternal age (years)	38.50±6.47
Total gonadotropin administered (IU)	2470.26±911.26
Estradiol level at hCG trigger day (pg/mL)	1935.24±1802.39
Interval between hCG and oocyte retrieval (hours)	35.34±0.42
***COS outcomes***	
Follicles (n)	15.40±11.77
Retrieved oocytes (n)	10.51±8.41
Oocyte yield (%)	68.87±20.09
MII Oocytes (n)	8.03±6.61
MI Oocytes (n)	1.12±1.56
GV Oocytes (n)	1.34±2.20
MII/oocytes (%)	77.69±20.99
MI/oocytes (%)	10.78±15.35
GV/oocytes (%)	11.28±15.56
***Laboratorial outcomes***	
Fertilization rate (%)	82.16±21.46
Embryos obtained (n)	6.64±5.08
High-quality embryos rate at day two (%)	25.74±24.60
High-quality embryos rate at day three (%)	48.59±28.40
Blastocyst rate (%)	47.60±32.48
Transferred embryos (n)	1.76±1.09
Endometrial thickness (mm)	10.68±2.28
***Clinical outcomes***	
Implantation rate (%)	31.83±41.36
Clinical pregnancy rate (%)	33.7
Miscarriage rate (%)	14.0

FSH: follicle stimulating hormone;

hCG: human chorionic gonadotropin;

IU: international units

The total dose of gonadotropin administered negatively affected the number of MI (*p*=0.004), and GV oocytes (*p*=0.029). Higher estradiol concentration at hCG day was correlated to higher number of MI (*p*<0.001), and GV (*p*<0.001) oocytes. The interval between hCG trigger and oocyte retrieval was not correlated to the number or rate of immature oocytes (Table 2).

**Table 2:** Contributing factors to immature oocyte incidence (n=3,920)

	MI			GV		
	R^2^	β	*p*	R^2^	β	*p*
Total gonadotropin dose	0.042	-0.046	**0.004**	0.050	-0.035	**0.029**
Estradiol level at hCG trigger day	0.146	0.324	**<0.001**	0.155	0.342	**<0.001**
Interval between hCG and oocyte retrieval	0.042	-0.015	0.368	0.050	-0.014	0.385
	**MI/oocyte**			**GV/oocyte**		
	**R^2^**	**β**	***p***	**R^2^**	**β**	***p***
Total gonadotropin dose	0.001	-0.009	0.567	0.002	0.009	0.592
Estradiol level at hCG trigger day	0.001	0.015	0.491	0.003	0.034	0.107
Interval between hCG and oocyte retrieval	0.002	-0.025	0.135	0.003	-0.015	0.356

hCG: human chorionic gonadotropin;

R^2^: effect;

β: standardized regression coefficient.

* Data was adjusted for maternal age, maternal BMI and total FSH dose.

In patients undergoing GnRH agonist protocols, GV/oocyte rate was increased (*p*<0.001) in patients receiving rFSH in comparison to rFSH plus rLH. Likewise, in GnRH antagonist regimens, the rFSH plus rLH stimulus was correlated to lower GV/oocyte rate (*p*=0.042) in comparison to rFSH (Table 3).

**Table 3:** Effect of the pituitary suppression and COS protocol on incidence of immature oocytes

	GnRH agonist				GnRH antagonista		
	rFSH (n=658)	rFSH plus rLH (n=712)	*P*		rFSH (n=1,570)	rFSH plus rLH (n=980)	*p*
MI	1.45±0.10	0.38±0.68	0.119		1.13±0.03	1.12±0.05	0.928
GV	1.46±0.14	0.40±0.93	0.263		1.33±0.05	1.36±0.08	0.731
MI/oocyte	13.40±0.91	6.32±6.19	0.147		10.75±0.36	11.33±0.59	0.405
GV/oocyte	11.52±1.12	1.86±2.10	**<0.001**		11.01±0.36	5.93±5.40	**0.042**

GnRH: gonadotropin-releasing hormone;

FSH: follicle stimulating hormone;

LH: luteinizing hormone.

* Data was adjusted for maternal age, maternal BMI, total FSH dose

The incidence of MI and GV oocytes were negatively correlated with fertilization rate (*p*<0.001), high-quality embryos rates at days two and three (*p*<0.001), as well as blastocyst rate (*p*<0.001). The negative effect was also noted in the implantation (MI/oocyte *p*<0.001; GV/oocyte *p*=0.033) and pregnancy (MI/oocyte *p*=0.002; GV/oocyte *p*=0.013) rates (Table 4).

**Table 4:** Regression analysis of the association between immature oocytes rate and ICSI outcomes (n=3,920)

	MI/oocyte			GV/oocyte		
	R^2^	β	*p*	R^2^	β	*p*
Laboratorial outcomes*						
Fertilization rate	0.035	-0.096	**<0.001**	0.029	-0.059	**<0.001**
High-quality embryos rate at day two	0.014	-0.102	**<0.001**	0.008	-0.066	**<0.001**
High-quality embryos rate at day three	0.020	-0.090	**<0.001**	0.020	-0.087	**<0.001**
Blastocyst rate	0.073	-0.066	**<0.001**	0.071	-0.053	**<0.001**
Clinical outcomes**						
Implantation rate	0.059	-0.074	**<0.001**	0.056	-0.042	**0.033**
	**B**	**OR**	*P*	**B**	**OR**	*p*
Pregnancy rate	-0.011	0.989	**0.002**	-0.009	0.992	**0.013**
Miscarriage rate	0.010	1.011	0.220	0.006	0.944	0.418

R2: effect;

β: standardized regression coefficient;

B: binary regression coefficient, OR: odds ratio for regression coefficient.

* Data was adjusted for maternal age, maternal BMI, total FSH dose, estradiol level at hCG day and number of retrieved oocytes.

** Data was adjusted for maternal age, maternal BMI, total FSH dose, estradiol level at hCG day, number of retrieved oocytes, number of transferred embryos and endometrial thickness.

Discriminant analyses were conducted to examine which factors would predict the pregnancy success in ICSI cycles. Mother age, number of transferred embryos, and MI/oocyte rate were the variables with higher correlation within function. The discriminant function could correctly classified 66.8% of original cases, with a better prediction for negative pregnancy, of 94.1%. In this model, the MI/oocyte rate of cycles that resulted in clinical pregnancy was of 9.56±11.25% and that of negative pregnancy of 11.49±16.00% (*p*=0.002). A cut-off point has been established halfway MI/oocyte rates averages, at 10.5%.

Cycles with MI/oocyte above cut-off were correlated to higher response to ovarian stimulation, with higher number of follicles and oocytes (*p*<0.001), lower fertilization rate (*p*<0.001), lower number of obtained embryos (*p*<0.001) and lower rate of high-quality embryos at day two (*p*<0.001). Implantation rate was reduced (*p*=0.038) and pregnancy rate was almost two times lower (*p*<0.001) (Table 5).

**Table 5:** Descriptive analysis of patients’ demographics and ICSI outcomes by MI/oocyte cutoff.

	MI/oocyte ≤10.5% (n=2,399)	MI/oocyte >10.5% (n=1,521)	*p*
***Patient’s demographics***			
Maternal age (years)	36.10±4.82	35.77±4.41	**0.034**
Maternal BMI (kg/m^2^)	24.29±3.81	24.39±3.88	0.060
Paternal age (years)	38.54±6.60	38.41±6.26	0.530
Total gonadotropin administered (IU)	2477.61±986.76	2453.75±770.55	0.431
Estradiol level at hCG trigger day (pg/mL)	1869.45±1797.94	2060.45±1820.42	0.051
Interval between hCG and oocyte retrieval (hours)	35.34±0.41	35.34±0.43	0.733
COS outcomes*			
Follicles (n)	14.80±0.22	16.49±0.27	**<0.001**
Retrieved oocytes (n)	10.09±0.16	11.22±0.19	**<0.001**
Oocyte yield (%)	68.29±0.41	69.71±0.52	**0.034**
MII Oocytes (n)	8.74±0.42	6.97±0.52	**<0.001**
MI Oocytes (n)	0.37±0.02	2.28±0.25	**<0.001**
GV Oocytes (n)	1.39±0.37	1.27±0.45	**0.024**
MII/oocytes (%)	86.23±0.37	64.51±0.46	**<0.001**
MI/oocytes (%)	1.69±0.22	24.87±0.27	**<0.001**
GV/oocytes (%)	11.82±0.32	10.45±0.39	**0.007**
Laboratorial outcomes**			
Fertilization rate (%)	83.15±0.44	80.47±0.55	**<0.001**
Embryos obtained (n)	6.85±0.59	6.35±0.074	**<0.001**
High-quality embryos rate at day 2(%)	27.0±0.50	23.7±0.60	**<0.001**
High-quality embryos rate at day 3 (%)	49.20±0.60	47.70±0.70	0.130
Blastocyst rate (%)	47.8±0.70	47.5±0.80	0.767
Transferred embryos (n)	1.69±0.23	1.87±0.28	**<0.001**
Endometrial thickness (mm)	10.62±0.53	10.78±0.65	**0.046**
Clinical outcomes***			
Implantation rate (%)	32.24±2.37	30.54±0.70	**0.038**
Clinical pregnancy rate (%)	43.72	22.25	**<0.001**
Miscarriage rate (%)	13.69	14.79	0.651

hCG: human chorionic gonadotropin;

IU: international units.

* Data was adjusted for maternal age, maternal BMI and total FSH dose.

** Data was adjusted for maternal age, maternal BMI, total FSH dose, estradiol level at hCG trigger day and number of retrieved oocytes.

*** Data was adjusted for maternal age, maternal BMI, total FSH dose, estradiol level at hCG trigger day, number of retrieved oocytes, number of transferred embryos and endometrial thickness.

## DISCUSSION

During COS, supra-physiological environment induce multiple follicular growth and maturation of oocytes that, which under natural conditions, would regress. Oocytes retrieved are not only at different stages of nuclear maturity, but probably also cytoplasmic maturity. Our evidence suggests that mature oocytes derived from a controlled stimulated cohort with high incidence of maturation fail may have inefficient biological machinery and detrimental clinical outcomes.

The gonadotropin dose is crucial for appropriate regulation of paracrine factors that induce proliferation and differentiation of pre-antral follicles and promote oocyte developmental competence (Thomas *et al*., 2005; Santi *et al*., 2017). The duration of FSH elevation above a critical threshold level, rather than the height of the elevation of FSH for single dominant follicle selection, is a key point for the recruitment of follicles from the resting pool during COS (Messinis & Templeton, 1990; van der Meer *et al*., 1994; van Santbrink *et al*., 1995). In fact, here we showed that ovarian hyper stimulation with higher gonadotropin doses was associated with a more homogeneous cohort development, with lower number of MI and GV immature oocytes.

In our study it was possible to correlate the COS protocol with the incidence of immature oocytes, since COS with exclusively rFSH was associated with higher GV/oocyte rate in comparison to rFSH associated with rLH, in both GnRH agonist and antagonist pituitary suppression protocols. Pior to midcycle LH surge, which is responsible for final oocyte maturation and ovulation, the growing oocyte acquires the ability to undergo oocyte maturation while being exposed to pulsatile LH basal levels (Mehlmann, 2005; Conti *et al*., 2012; Celik *et al*., 2015; Coticchio *et al*., 2015). Thus, LH association to FSH during follicular development may promote a more physiologically environment, and can be a manner to improve oocyte maturation. Some authors observed that the presence of LH activity may have a positive effect on oocyte maturation (Selman *et al*., 2002; 2010; Pacchiarotti *et al*., 2007; Santi *et al*., 2017) and LH supplementation had been correlated to higher number of mature oocytes in COS protocols for anovulatory patients (Burgués & Spanish Collaborative Group on Female Hypogonadotrophic, 2001; Raju *et al*., 2013) or patients with poor ovarian response (Papaleo *et al*., 2014).

Higher estradiol concentration at hCG day was highly associated with the number of immature oocytes, in accordance to previous reports (Dor *et al*., 1992; Suchanek *et al*., 1994). Although the time interval between hCG trigger and oocyte retrieval is a well-established fundamental parameter to the retrieval of mature oocytes (Raziel *et al*., 2006; Son *et al*., 2008; Wang *et al*., 2011), we noticed a small variance in the time interval comparing different cycles, so it was not possible to correlate this factor with immature oocyte incidence.

The rate of both MI and GV oocytes negatively impacted on the fertilization of the MII oocytes from the same cohort. In fact, increasing incidence of mature oocytes has been correlated with increasing fertilization rate (Halvaei *et al*., 2012; Kahraman *et al.,* 2017). The oocyte competence depends on a multiplicity of factors; one of them is the ability to generate normal Ca^2+^ oscillation response induced by sperm penetration, that develops during the final stages of oocyte growth and maturation and is essential for promoting oocyte activation and fertilization initiation; thereafter MII oocytes that did not reach fully completed cytoplasmic maturity could have impairment in the acquisition of developmental competence (Cheung *et al*., 2000)

The fertilization of mature oocytes derived from cycles with heterogeneous follicular development can lead to abnormal early embryo development, with embryos with significantly more cleavages and lower blastocyst quality (Halvaei *et al*., 2012; Kahraman *et al*., 2017). Correlations of immature oocyte rates with lower cleave-stage quality and lower blastocyst formation were observed. However, the correlation of immature oocytes and embryo formation was not observed by others (Son *et al*., 2008).

The lower fertilization, high-quality embryo and blastocyst rates impacted directly on the implantation and pregnancy rates. We could establish a cut-off based on discriminant analysis of MI/oocyte rate of 10.5% for the prediction of pregnancy outcome. We observed that cycles with rate higher than 10.5% were correlated to higher response to ovarian stimulation, poor embryo development and almost two times lower pregnancy rate.

In conclusion the present study suggests that immature oocyte incidence is affected by the COS protocol and that immature oocyte incidence negatively impacts ICSI outcomes of mature oocytes from the same cohort. Our findings also demonstrated the importance of COS protocol and pituitary suppression type on the outcomes of ICSI cycles. And finally, cycles with lower than 10.5% of MI/oocyte should be closed followed.
